# Social and Poor Law Problems

**Published:** 1908-03-21

**Authors:** 


### SOCIAL AND POOR-LAW PROBLEMS.

### A CURE FOR STAMMERING.

Among the minor ills of life, stammering takes a prominent place. If it does not cut a man off from a career, as do blindness or deafness, it interferes with his progress at many points, and at least makes him feel awkward among his fellows. Mr. A. C. Schnelle, of 119 Bedford Court Mansions, London, W.C., who was himself a stammerer once, has given his attention to the subject of stammering, and has devised a method of cure. With a view to studying Mr. Schnelle's treatment and its success, we have visited his classes more than once, and can say, from the progress made by the pupils in a few weeks, that he really does cure stammering. Under his instructions stammerers lose in a few days the absolute stutter which impeded their articulation, though one notices that they speak with great carefulness, and their talking or reading is slow and less automatic than that of ordinary persons. After a fortnight or so, these signs of care disappear, and the speech becomes quicker and less obviously an effort. A man who spoke no worse might easily go about among his fellows without his defect being noticed. And in the end the defect vanishes, and the ex-stammerer speaks exactly like other people. With pupils who come to him daily Mr. Schnelle generally effects a cure in any time up to ten weeks, while with resident pupils, who are continually under his supervision, the cure is completed in about four weeks. Of course the length of time required for a complete cure depends to some extent on the pupil's intelligence and application, and here, perhaps, adult pupils, who are taking the course on their own initiative, have the advantage over younger ones, who are less conscious of the handicap which stammering may cause in their future life. But the training itself, by curing the slovenliness of speech which lies at the back of stammering, has a certain moral effect, and brightens up the pupil in other ways. Mr. Schnelle charges by the cure, and not by the time taken to effect it. Having watched the progress of pupils from the beginning of their course until they were well advanced, and having hear[illegible] several of the more advanced speak, read, and recite, w[illegible] can say that Mr. Schnelle effects a cure of stammering, an[illegible] sends his pupils out into the world well able to hold thei[illegible] own with their neighbours in the matter of clear an[illegible] fluent speech.

### DRUGGED SWEETS.

Mr. Scott Elder, the chief inspector under the Foo[illegible] and Drugs Act for the County of Durham, has had a[illegible] analysis made of some sweets bought in the county, an[illegible] finds that in some there are drugs and in others alcoho[illegible] It cannot be said that there is any deception about them[illegible] for the former are frankly sold under the name of "chloro[illegible] dyne gums" or "chlorodyne lozenges." Each gum wa[illegible] found to contain 0.15 minim of chloroform, an[illegible] each lozenge 0.06 of the same. The accepted mini[illegible] mum dose of chloroform which can be given inter[illegible] nally is, according to the British Pharmacopœia one minim, so that if anyone ate seven of these gums h[illegible] would absorb one minimum dose of chloroform. Th[illegible] sweets are sold at a penny an ounce, and the ounc[illegible] averages twenty-one sweets, so that consuming an ounc[illegible] of these is equal to taking three such doses. Ye[illegible] these sweets can be freely sold to children. Other sample[illegible] which Mr. Scott Elder analysed were called "chocolat[illegible] liqueur beans" and "rum and butter sweets," and i[illegible] each of these were to be found minute traces of alcohol. The inspector does not think that this was actually addec to the sweets, but it was present in the flavouring. It is to be regretted that there is a great demand for these sweets among children, and the danger is perhaps all the greater that they know that it is the alc[illegible]ol they like, for they ask for them under the name of "whisky chocolates." There is, of course, an insidious danger in getting to like the taste of alcohol without knowing what it is, but the ignorance at least puts a barrier in the way of obtaining the stimulant in other forms. But children who buy "whisky chocolates" in their youth will have no difficulty in finding out what it is they crave for when they are older.

